# Social Cognitive Skills in Electric Vehicle Sales: Understanding Empathy, Trust, and Decision-Making in Sustainable Mobility Markets

**DOI:** 10.3390/bs15121681

**Published:** 2025-12-04

**Authors:** Sergio Escobar-Miranda, Luis Ballesteros-Sánchez

**Affiliations:** Departamento de Ingeniería de Organización, Administración de Empresas y Estadística, Escuela Técnica Superior de Ingenieros Industriales, Universidad Politécnica de Madrid, 28040 Madrid, Spain; luisignacio.ballesteros@upm.es

**Keywords:** social cognition, decision-making, empathy, trust, electric vehicles, salesperson competencies, consumer behavior

## Abstract

The transition to electric mobility requires salespeople to go beyond technical expertise and develop advanced social–cognitive skills that shape consumer decision-making. This study examines how empathy, perspective-taking, and trust influence interactions between salespeople and potential buyers of electric vehicles (EVs). Through a systematic literature review and bibliometric analysis, we identify the key cognitive and emotional competencies that enable sales professionals to interpret customer intentions, manage uncertainty, and guide rational yet emotionally influenced purchase decisions. Findings suggest that successful EV sales rely on understanding consumer beliefs about sustainability, risk, and technology, as well as on the salesperson’s ability to align messages with these cognitive frames. Based on this analysis, we propose a competency development framework that emphasizes empathy-driven communication, adaptive reasoning, and the integration of social cognition into training strategies. This perspective contributes to the broader understanding of how social–cognitive processes affect human judgment and decision-making in the emerging electric vehicle market.

## 1. Introduction

Understanding human judgment and decision-making in the context of sustainable consumption increasingly depends on social–cognitive processes such as empathy, trust, and perception of others’ intentions. In sales interactions, these mechanisms shape how individuals interpret persuasive messages, evaluate technological innovations, and form purchase intentions. On the other hand, electric vehicles (EVs) are increasingly recognized as an important component of the global effort to achieve the Sustainable Development Goals (SDGs) set by the United Nations (Omahne et al., 2021). The drive towards EV adoption not only redefines the nature of the product but also transforms market dynamics and, consequently, the seller’s role (Lam & Mercure, 2020). Although electric vehicles (EVs) are a potential solution for decarbonizing the transport sector and contribute to the fulfillment of the SDGs, their social acceptance is a key factor that warrants further research (Omahne et al., 2021). This paper analyzes the key competencies sales professionals must possess to succeed in the emerging electric vehicle market. This sector, characterized by disruptive technology, new forms of consumption, and customers with different expectations, requires a reconfiguration of commercial strategies and an adaptation of salespeople’s skills (Totlani, 2023). Defining these skills is especially important, given that information on the role and competencies of EV marketers is almost nonexistent. Therefore, there is a significant gap in practical knowledge that could contribute to the adoption of EVs by developing EV salesperson competencies.

The objectives of this work are as follows: First, we identify, through a literature review (scoping review), the competencies and skills that salespeople across different industries should possess in order to determine their applicability to the EV industry. This objective contributes to the study of competencies, as an updated literature review is necessary to advance knowledge of salesperson competencies in general. The second objective of this article is to create a framework for developing key competencies and skills for EV salespeople. This point is critical given that EV adoption has experienced stagnation in several regions over the past year (Irle, 2024). This fact, coupled with the disparity in EV adoption across regions, signifies a growing problem for EV sales. Thus, this study aims to address that problem by developing key competencies for EV salespeople.

## 2. Background

### 2.1. Adoption of Electric Vehicles

The transition to sustainable mobility is a global imperative in the fight against climate change (Cruz-Jesus et al., 2023), and electric vehicles (EVs) are a key component of this transition (Adnan et al., 2017). Therefore, addressing technological, financial, and infrastructure barriers, while promoting their environmental benefits, is critical to accelerating the transition to electric mobility (Pamidimukkala et al., 2023). To maximize its impact, a holistic approach is needed that includes the aforementioned dimensions: infrastructure, public policies, environmental awareness, and user considerations regarding green identity. The adoption of EVs represents not only a technological breakthrough but also a cultural and social shift towards more environmentally friendly practices (Pamidimukkala et al., 2024).

On the other hand, innovative features of EVs, such as technological compatibility and relative advantages, along with perceptions of risk, significantly influence purchase intention. Fashion consciousness, environmental consciousness, and price consciousness are lifestyle aspects that modulate the relationship between innovative features and risk perception (Xie et al., 2022).

### 2.2. The Role of the Dealership

The dealership has been fundamental in the marketing of automobiles. Their function was centered on product presentation, price negotiation, and after-sales management. However, the irruption of electric vehicles, together with the increasing digitalization of the market, is modifying this scheme (Genzlinger et al., 2020). The technological complexity of the product, the need for advice on charging infrastructure, and the new online purchasing options demand a more specialized salesperson profile with skills that go beyond the traditional ones (Anderson et al., 2022).

Hence, it is clear that the EV sales process is a significant aspect in the adoption and penetration of EVs in the market, and in that aspect, car dealers are key players in this process, since it is in their facilities that the interaction of sales staff with the end customer takes place (Matthews et al., 2017; Tromaras et al., 2017). Although technology, infrastructure and government policies are important, the personal shopping experience between the potential buyer and the salesperson can be a decisive factor in convincing consumers, as dealers and their staff must carry a very clear idea of the goodness of EVs and their technology, and their approach to the sales process can significantly influence the consumer’s perception and purchase decision (Matthews et al., 2017).

The academic literature has approached the transformation of the automotive sector from various perspectives.

Muratori et al. (2021) argue that the automotive sector has moved towards a sustainable model through the integration of electric vehicles (EVs) and the convergence with renewable energies. This transformation is materialized, according to the authors, through new mobility models, such as shared transport, which optimizes vehicle use and reduces the need for individual ownership, and automation, which promises greater energy efficiency and road safety. The electrification of transportation, driven by advances in batteries and the expansion of charging infrastructure, reduces dependence on fossil fuels and greenhouse gas emissions. Furthermore, the author stresses that synergy with renewables, through smart charging and the integration of EVs into the grid, enables more efficient energy use and better management of intermittency from renewable sources.

On the other hand, Ahmadi et al. (2023) state that the advent of Industry 4.0, with its emphasis on digitization and interconnectivity, has revolutionized the way automakers interact with consumers. No longer relying solely on traditional advertising and interaction with salespeople, companies are leveraging data analytics, online platforms, and personalized digital experiences to connect with potential buyers. In addition, the growing focus on sustainability influences marketing narratives, highlighting environmentally friendly features and manufacturing processes to appeal to eco-conscious consumers. This shift towards digital, data-driven, and sustainable marketing practices is essential for automakers to remain competitive amid evolving consumer demands and technological advances.

Precisely with respect to technological advances, Sterk (2024) concludes that this evolution has transformed the automotive sector by driving the development of connected vehicles. This fact has enabled data collection, opening the door to new business models based on smart services such as predictive maintenance and personalized insurance. In addition, connectivity has facilitated the development of functions such as traffic assistance and parking, changing consumer preferences, and requiring traditional manufacturers to adapt. At the same time, increasing digitalization poses challenges for privacy and data sharing, requiring a balance between monetization and the protection of user information.

### 2.3. The Marketer’s Role and Competencies

In this new scenario, the salesperson must evolve from a transactional to a consultative role (Paesbrugghe et al., 2020). The role of the salesperson has undergone a continuous evolution, reflecting changes in market dynamics and technological advances. From the traveling salesman of antiquity to today’s sophisticated e-commerce strategies, the core function remains the same: to connect the product with the consumer. Historically, in the automotive industry, this connection was forged through a predominantly transactional approach (Corsaro & Maggioni, 2022). The salesperson, often operating within the confines of a dealership, focused on highlighting product features, negotiating price, and facilitating after-sales service. This model, effective in an era of relative product homogeneity and limited consumer access to information, positioned the salesperson as a knowledge gatekeeper (Singh et al., 2015).

However, the advent of the internet and the rise of e-commerce platforms have fundamentally altered the consumer journey. Shoppers now arrive at the dealership armed with extensive product knowledge gleaned from online reviews, comparisons, and virtual showrooms (Bacher & Manowicz, 2020). This shift has diminished the salesperson’s traditional informational advantage, necessitating a reassessment of their role and skill set. The transition to electric vehicles further accelerates this transformation. The inherent complexity of electric vehicle technology, coupled with concerns around range, charging infrastructure, and battery life, demands a more consultative and advisory approach. The salesperson must now navigate a landscape of changing consumer expectations, acting not just as a conduit for transactions but as a trusted advisor, able to address complex technical questions and provide customized solutions for individual mobility needs. This evolution requires a deeper understanding of the technology, the market, and the changing needs of the EV consumer (Zarazua de Rubens et al., 2020).

Ultimately, this paper seeks to identify the competencies and soft skills essential to the success of electric vehicle (EV) marketers in the context of the evolving automotive industry. Considering the unique characteristics of EVs, changing consumer expectations, and new marketing tools, we will analyze the transition from a traditional sales approach based on technical specifications to a consultative model. This new model prioritizes a deep understanding of customer needs and provides the keys to developing sales personnel’s skills and competencies, thereby impacting the EV industry and marketing. Therefore, we will explore how the salesperson’s role adapts and redefines itself to drive sustainable industry growth and customer satisfaction in this emerging paradigm.

### 2.4. Social–Cognitive Skills

Social–cognitive skills are psychological abilities that help individuals understand and respond to others’ emotions, intentions, and viewpoints. Recent studies show that these skills, such as perspective-taking, interpersonal accuracy, and the inference of mental states, are vital for effective social interactions, especially in complex and uncertain professional settings, where perspective-taking ability is associated with greater social ability (McGarry et al., 2021). In decision-making scenarios where customers encounter technological or informational uncertainties, strong social–cognitive skills enable professionals to identify concerns early, tailor their communication, and foster interactions that promote understanding and reduce cognitive effort (Falk & Bassett, 2017).

On the other hand, empathy is increasingly seen as a vital part of social–cognitive skills, especially in roles requiring interpersonal influence and delicate emotional communication. Unfortunately, the varying definitions of empathy have adversely affected both research and practice (Cuff et al., 2016). In this sense, modern psychological theories differentiate between cognitive empathy (viewing things from another’s perspective) and affective empathy (feeling with another), each playing a unique role in effective interactions and resolving issues. Thus, it is relevant to understand that real-life empathic goals span a broad spectrum, sometimes involving worsening the targets’ affect or opposing their wishes to enhance their well-being, which can be effectively linked to interpersonal emotion regulation (Zaki, 2020). Accordingly, cognitive empathy particularly enhances customer communication, fosters relational harmony, and helps create shared understanding during complex service interactions. These insights show that social–cognitive abilities are not only measurable but also cultivable through structured training and reflection.

Trust formation, a key process linking social–cognitive skills to behavioral results, has gained renewed interest in recent organizational studies. Trust develops when individuals see their interaction partner as both capable and benevolent, perceptions that are heavily influenced by social–cognitive skills such as empathy, interpersonal sensitivity, and clear communication. Research shows that trust influences both intra- and interpersonal functioning and overall well-being, with these relationships being mediated by the mind (Petrocchi et al., 2021). Other recent findings also reveal that psychological safety precedes trust and collectively increases individual job satisfaction (Mitterer & Mitterer, 2023), which helps explain why employees with higher social–cognitive skills are more adept at engaging in adaptive communication, building stronger customer relationships, and reducing uncertainty in high-stakes decisions. This finding underscores the importance of social–cognitive competence as a psychological foundation for trust development in modern service and sales environments.

### 2.5. Research Gap

While there is a growing body of literature on electric vehicle marketing and, separately, on the competencies required for selling in general, there is a glaring research gap at the intersection of these fields, specifically regarding the competency mix (both hard and soft), the ideal salesperson profile, and electric vehicle marketing. This gap limits the understanding of how specific salesperson skills, in conjunction with their personal and professional profiles, affect the effectiveness of marketing strategies in this emerging niche market. Consequently, the development of education and training programs that adequately prepare sales professionals to meet the challenges and opportunities presented by electric vehicle marketing is hindered.

The paucity of studies that integrate competencies, salesperson profiles, and marketing for electric vehicles has significant implications for both the automotive industry and academic research. For the industry, this lack of knowledge hinders the optimization of salesperson selection, training, and performance evaluation processes, thereby impacting sales efficiency and customer satisfaction. From a research perspective, this gap represents a fertile area for exploration. Future studies should focus on identifying the most relevant soft and hard competencies for success in electric vehicle sales, as well as on defining the ideal salesperson profile, accounting for variables such as prior experience, academic background, and personality characteristics. It is also valuable to investigate how these competencies and profiles relate to different marketing aspects and to the different segments of electric vehicle consumers.

## 3. Methodology

It is important to note that systematic reviews provide a clear, rigorous framework for searching, selecting, and analyzing documents (Tawfik et al., 2019). In this regard, the present study uses a qualitative, descriptive, and secondary research method, relying on a systematic literature review to thoroughly analyze the social cognitive skills in EV sales (Hernández Sampieri et al., 2014). In other words, this systematic literature review aims to analyze scholarly research on social cognitive skills in EV sales, highlighting the benefits, costs, and opportunities associated with adopting technological innovations in the industry.

This study examines participation in sales-innovation and technological-transformation policies within the EV sector. It considers scientific articles, technical documents, reports, and theses that offer empirical evidence or insights on social cognitive skills in EV sales. The review covers publications from 2009 to 2025, capturing current technology, regulations, and sales practices. The literature search was limited to the Scopus and Web of Science (WoS) databases. These sources were chosen for their rigorous indexing, broad disciplinary coverage, and inclusion of high-impact, peer-reviewed journals. Their organized metadata and citation data ensure reliable and reproducible information for both quantitative and qualitative analysis. This approach aligns with standard practices in systematic and bibliometric research, ensuring methodological consistency and comparability with other studies.

To perform this systematic literature review, the following is an example of a Boolean algorithm used in the search process:

(“sale” OR “sell”)

AND

(“elect* vehic*” OR “EVs”)

AND

(“BEVs” OR “electric mobility”).

For filtered databases, keyword co-occurrence maps were created to identify thematic branches and connections between articles. The proximity of keywords on the map indicates a strong relationship, while greater distances suggest weaker links (van Eck & Waltman, 2010). Thematic analysis focused on identifying key recurring themes linking research on social cognitive skills in EV sales. The most important terms were recognized by their high co-occurrence, visualized as larger nodes in the bibliographic map (Garcés et al., 2025). Subsequently, interactions or clusters were examined to highlight the most significant relationships between themes and to understand the research field’s structure better. For the results analysis, the selected articles were coded and examined, with thematic analysis supported by word clouds, conceptual networks, and a coding matrix to help compare the findings and produce cross-referenced evidence.

## 4. Skills or Competencies for Selling Electric Vehicles

### 4.1. Search Process

Based on the previous methodology, a co-occurrence analysis of these three research areas is presented below:

- Competencies and skills (Keywords: competen* OR skill* OR abilit* OR trait*).
- Sales or Marketing (Keywords: sale* OR sell*)
- Marketing of Electric Vehicles (Keywords: “elect* vehic*” OR “EVs” OR “BEVs” OR “electric mobility”)

**Figure 1:**
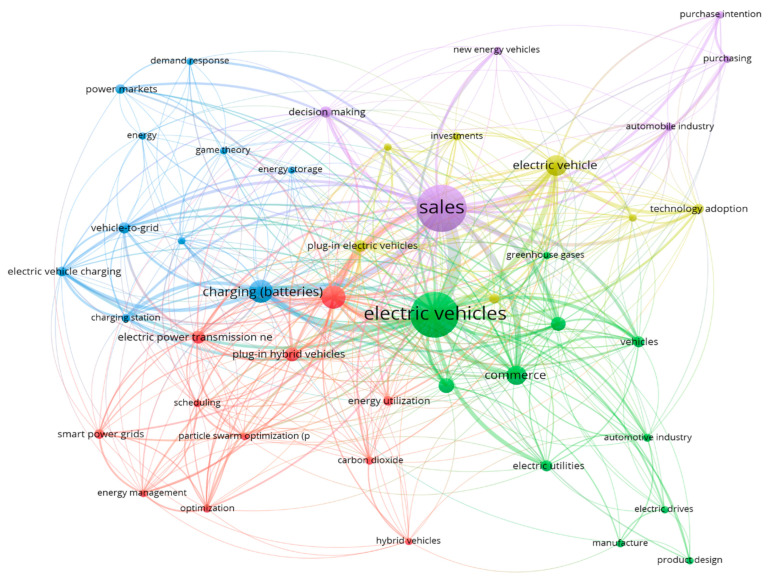
Co-occurrence analysis of 3 variables (Scopus and WoS).

The choice of these keywords was made as broad as possible to ensure an exhaustive search and the possibility of results after analysis. The search for topics was conducted in both Scopus and Web of Science. However, in both cases, we found publications that used a different approach from the one required in the present research, as shown in Figure 1.

As we can see, although there is a genuine connection between sales and electric vehicles, no branch of this graph mentions skills, competencies, or capabilities. The articles are mainly concentrated on:

- Incentive Measures and Subsidies for Acquisition.
- Factors influencing purchase intention.
- Electric vehicle technologies and benefits
- Manufacturing engineering processes
- Environmental impact of EV adoption.

### 4.2. Proposed Model: Dual Approach Analysis

Given the above, an analytical approach is proposed that examines two separate dimensions: on the one hand, competencies and skills, along with the general profile of the successful salesperson in today’s industries, and on the other, the particularities of electric vehicle sales and the specific demands of this market. This dual analysis allows, firstly, to establish a solid base of competencies and skills considered essential for success in sales, regardless of the product. Secondly, it facilitates the identification of the specific needs and challenges in marketing electric vehicles, such as technical product knowledge, understanding of new technologies, and the ability to address consumer concerns in this regard.

The conjunction of both analyses, salesperson competencies and skills, and the particularities of the EV market, provides the basis for projecting the profile of the ideal salesperson in this emerging sector. By contrasting the general competencies of the salesperson with the specific demands of electric vehicle marketing, it is possible to identify areas of convergence and areas requiring development or specialization. This process not only defines the technical skills and knowledge required but also helps understand how traditional competencies and skills need to be adapted and enhanced for optimal performance in this context. For example, active listening skills, fundamental in any sales process, take on a new dimension in the marketing of electric vehicles, where the salesperson must understand the customer’s specific needs and concerns related to electric mobility, such as anxiety about autonomy or the availability of charging points. In short, integrating both analyses allows building a comprehensive profile of the electric vehicle salesperson, combining essential sales skills with specialized knowledge of the product and the market.

## 5. Skills and Competencies of Salespeople in Different Industries

This section presents a review of the academic literature on the skills and competencies required for successful B2C salespeople. A systematic search of Web of Science and Scopus was conducted, selecting scientific articles, book chapters, and conference proceedings published in English since 2010. Only studies presenting quantitative or qualitative results on specific salespeople skills, competencies, or traits were included, discarding research on general marketing, market analysis, case studies without focus on individual salespeople skills, opinions, retractions, or studies on B2B, online, or other non-B2C sales channels. This approach allows for an in-depth analysis of the key competencies for successful B2C sales performance, offering a focused perspective on the development and optimization of human capital across different industries. The total number of articles analyzed is provided in Appendix A.1 of this article.

### 5.1. Search Strategy for Salespeople

For the search strategy, our objective is to define the skills, competencies, and traits that successful salespeople have. For that, we used a search strategy that limited the results to only articles that discuss the competencies, skills, and traits of salespeople.

- Publication indexed in Web of Science or Scopus.
- Search terms and Boolean operators:
  - Web of Science: TI = (sale* OR sell*) AND TI = (competen* OR skill* OR abilit* OR trait*)
  - Scopus: (TITLE ( “Sale*” OR “Sell*” ) AND TITLE ( competen* OR skill* OR abilit* OR trait* ) ) AND PUBYEAR > 2009 AND PUBYEAR < 2025 AND ( LIMIT-TO ( LANGUAGE, “English” ) ) AND ( LIMIT-TO ( DOCTYPE, “ar” ) OR LIMIT-TO ( DOCTYPE, “cp” ) OR LIMIT-TO ( DOCTYPE, “ch” ) ) )
- Year of publication greater than 2010
- Language English
- Is it an original article, conference paper, or book chapter

For both searches, we limited the results to articles with a publication year greater than 2010, in English, and that were original articles (journal articles), conference proceedings, or book chapters. The reason we limited the search to results with those words in the title is that, when we expanded the search to include the search words in the abstract or full text, both databases returned up to hundreds of thousands of results. This aspect is because the words “sales” and “skills” (for example) are applicable across many disciplines, and the results were inconsistent with our objective of examining only salespeople’s competencies, skills, or traits.

### 5.2. Inclusion Criteria for Salespeople

The following are the main criteria considered for inclusion analysis:

- Studies since 2010 in the English language.
- Studies that present results on salesperson skills, competencies, or traits.
- Studies that indicate the industry in which the study was conducted in relation to direct selling, business-to-customer (B2C).

### 5.3. Exclusion Criteria for Salespeople

The following are the main criteria considered for exclusion analysis:

- Opinion studies, retractions, conference proceedings with abstract only.
- Studies that do not specify specific skills, competencies, or traits of salespeople.
- Studies that do not specify the industries in which they were conducted.
- Studies on B2B (business-to-business) sales, online sales, or sales through channels other than B2C.

### 5.4. Flowchart for Salespeople

**Figure 2:**
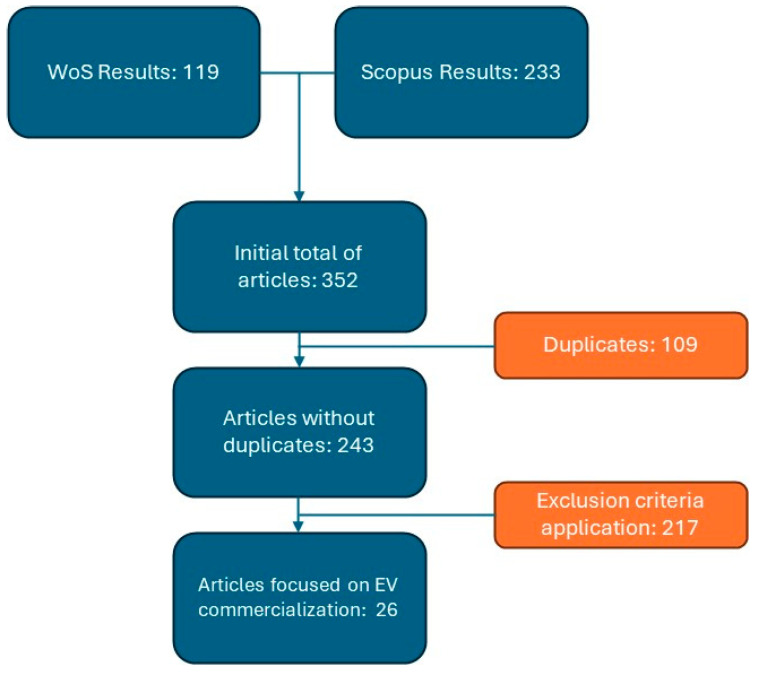
Flowchart of inclusion and exclusion of articles in the literature review for salespeople.

Based on the previous inclusion and exclusion analyses, the flowchart shown in Figure 2 was selected for the literature review process.

### 5.5. Co-Occurrence Analysis (Sales Force + Competencies)

**Figure 3:**
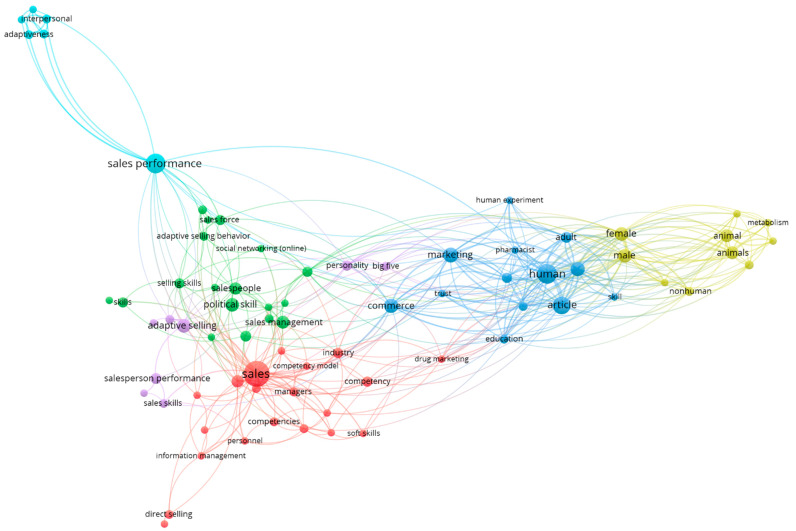
Co-Occurrence Analysis: SALES + COMPETENCIES.

In addition, we present the co-occurrence analysis of both databases, where we can clearly see the key issues addressed by these studies (Figure 3), which will be analyzed in later paragraphs:

### 5.6. Analysis and Discussion of Sales Force Competencies and Skills

Sales force competencies and skills play a valuable role in the business performance of organizations. By analyzing 26 relevant publications, it is possible to identify common patterns and key competencies that emerge in the literature on the subject.

One of the most recurrent findings is the importance of interpersonal skills, which were highlighted by the publications of Agnihotri et al. (2012, 2016). In their studies, they emphasize that empathy and the ability to build relationships are critical to sales success, especially in competitive markets such as India and Brazil. These skills enable salespeople to effectively connect with customers, understand their needs, and build trust, which inevitably translates into higher customer satisfaction and, ultimately, better sales results.

Another aspect highlighted in various publications is the need for specific skills tailored to particular sectors. Ahmad et al. (2010) note that, in the telecommunications sector, sales skills are especially critical for managing the complexity of the products and services offered. This finding is complemented by the studies of Kimura et al. (2019) and Kazén et al. (2013), who argue that the adaptation of skills to the demands of the sales context can be determinant for success. Here, there is consensus that skills should evolve and be tailored to the sales environment, enabling better resonance with consumers.

Mindfulness is also mentioned as a central competency, particularly in the studies of Charoensukmongkol and Pandey (2021). This finding is groundbreaking, as it suggests that a salesperson’s ability to be fully present in the interaction may be as important as his or her technical skills. Mindfulness can improve communication quality and the effectiveness of customer interactions, leading to positive sales outcomes.

In addition, several studies, including those by Das et al. (2018) and Hsiao (2012), suggest that analytical thinking and politeness are competencies that not only improve sales performance but also enhance real-time problem-solving skills. A salesperson’s ability to analyze situations and adapt quickly to customer needs is essential, especially in high-pressure situations. These skills allow for a more holistic view of the customer experience, which is effective in closing deals.

At the motivational and personality levels, the findings of Lilford et al. (2014) highlight that traits such as conscientiousness and extroversion are vital for success in sales, a point also supported by the research of Mostafa and Kasamani (2022). A salesperson’s personality can influence his or her ability to connect with the customer, as well as his or her resilience and persistence in challenging environments. These traits, along with intrinsic motivation, determine a salesperson’s level of commitment and ability to meet or exceed sales goals.

Effective communication and listening skills are another area of consensus in the analyzed publications. Omar (2017, 2018) highlight that clear communication and active listening are essential elements in sales interactions. Understanding customer concerns through active listening enables salespeople to tailor their approach and respond more effectively to customer needs.

Finally, it is noted that sales competencies are not static. Studies by Phongkhajeerathibha et al. (2023) and Rantala et al. (2020) emphasize the need for continuous training and the identification of new competencies as the market evolves. In an ever-changing sales environment, adaptability and continuous learning are essential to remain competitive and successful.

## 6. Complexity of Electric Vehicle Marketing and Sales

This second section presents a review of the academic literature on the particularities of electric vehicle (EV) marketing and sales. To ensure the review’s relevance and focus, a search strategy was implemented in Web of Science and Scopus, focusing on original articles, conference papers, and book chapters published in English since 2010. The selection was based on keywords related to commercialization, the sales process, and key factors for EV adoption, excluding studies focused on government incentive policies, technical or engineering aspects, and environmental impact. This approach allows for an in-depth analysis of the specific challenges and opportunities faced by vendors in this emerging market, offering a focused perspective on commercial and consumer dynamics. The total number of articles analyzed is provided in Appendix A.2 of this article.

The search strategy, along with inclusion and exclusion criteria, is described as follows.

### 6.1. Search Strategy for EV Marketing and Sales

We used a search strategy that limited results to articles that discuss electric vehicles, electric mobility, adoption, sales, and selling.

- Publication indexed in Web of Science or Scopus.
- Search terms and Boolean operators:
  - Web of Science: (TI = (“electric vehicl*” OR “EVs” OR “BEVs” OR “electric mobility”) AND (TI = (adoption)) AND (TS = (“sale*” OR “sell*”)))
  - Scopus: (TITLE (“Electric Vehicl*” OR “EVs” OR “BEVs” OR “Electric Mobility”) AND TITLE-ABS-KEY ( “SALE*” OR “SELL*” ) AND TITLE ( adoption ) ) AND PUBYEAR > 2011 AND PUBYEAR < 2025 AND ( LIMIT-TO ( DOCTYPE, “ar” ) OR LIMIT-TO ( DOCTYPE, “cp” ) OR LIMIT-TO ( DOCTYPE, “ch” ) ) AND ( LIMIT-TO ( LANGUAGE (“English”) ) )
- Year of publication greater than 2010
- Language English
- Document is an original article, conference paper, or book chapter.

### 6.2. Inclusion Criteria for EV Marketing and Sales

- The research focuses on electric car marketing, sales process, and/or key factors in adoption.

### 6.3. Exclusion Criteria for EV Marketing and Sales

- Deals with government subsidy and incentive policies.
- Technical, industrial, or engineering issues
- Addresses the environmental impact of EV adoption.

### 6.4. Flowchart for EV Marketing and Sales

**Figure 4:**
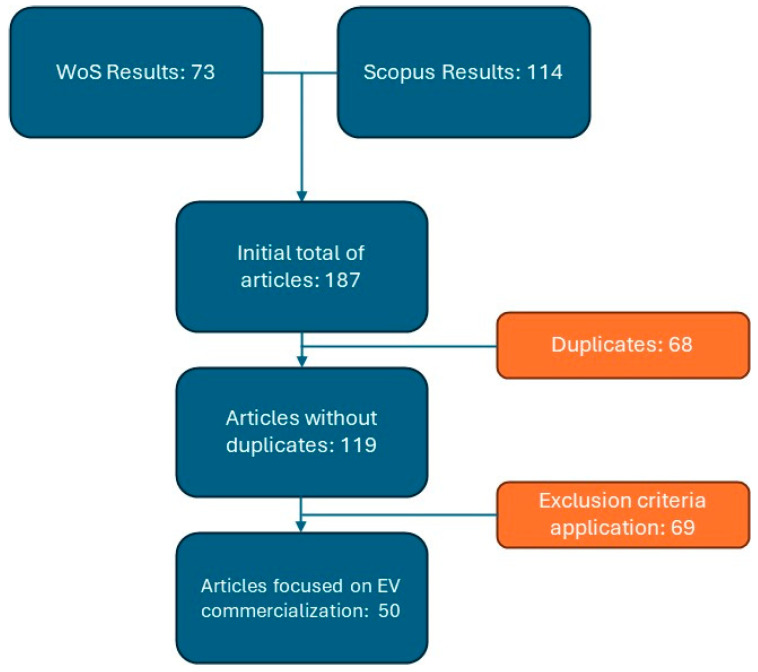
Flowchart of inclusion and exclusion of articles in the literature review for EV marketing and sales.

Based on the previous inclusion and exclusion analyses, the flowchart shown in Figure 4 was selected for the literature review process.

### 6.5. Co-Occurrence Analysis for EV Marketing and Sales (Sales Staff + Competences)

**Figure 5:**
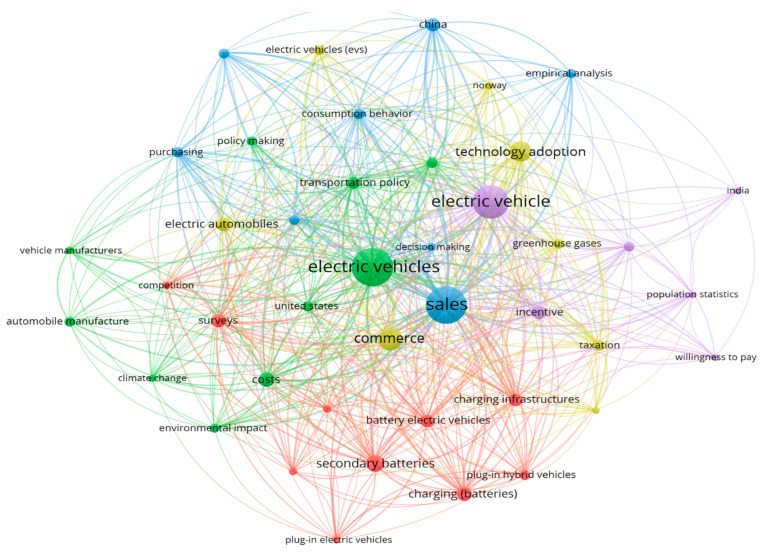
Co-Occurrence Analysis: EV + Sales.

In addition, we present the co-occurrence analysis of both databases, where we can clearly see the key issues addressed by these studies (Figure 5), which will be analyzed in later paragraphs:

### 6.6. Analysis and Discussion on the Dynamics of Purchase and Adoption of Electric Vehicles

In Appendix A.2, we find the details of all the reviewed articles, together with a summary of the main findings. The analysis of this literature reveals several recurring and problematic themes that are critical to understanding the new paradigm facing salespeople in this sector.

First, a central theme that emerges from the findings is the high initial cost associated with electric vehicles (EVs). According to Abas and Tan (2024), this represents a significant barrier to widespread adoption. This finding is supported by Anosike et al. (2023), who note that the high initial price creates considerable uncertainty about the long-term value of EVs. This negative perception can hinder consumers’ willingness to invest, posing a challenge that marketers must address through educational and consultative strategies. The customer experience must be transformed through an approach that prioritizes demonstrating the long-term savings and environmental benefits that EVs offer.

In addition to cost, the limited range of EVs is another recurring finding. This concern, noted by Abas and Tan (2024), is intimately connected to the need for marketers to understand the product’s technical characteristics. The range of an EV is a significant concern for consumers. As indicated by Bjerkan et al. (2016), limited autonomy can generate apprehensions in potential buyers, who fear not having enough charging options during their commutes. Barter et al. (2015) also reinforce this view by noting that an understanding of autonomy and its manageability are paramount for the salesperson to influence consumers’ purchase decisions effectively. Salespeople must be equipped with robust technical information to effectively answer these questions and compare with internal combustion vehicles, indicating a shift in the skill profile required of today’s salesperson.

Another key finding is the reflection on the charging infrastructure. Mpoi et al. (2023) and Munshi et al. (2022) agreed that fiscal incentives, such as purchase tax exemptions, play a key role in EV adoption. However, the success of these policies also depends on the availability and accessibility of charging infrastructure. This aspect highlights the need for vendors not only to focus on the act of selling, but also to offer comprehensive solutions that address consumers’ charging needs for their vehicles. This includes information on loading locations, associated costs, and times required for loading.

Effective communication is also critical in this new paradigm. Research has shown that consumers often prioritize knowledge about the benefits and limitations of EVs from marketers. According to Lim et al. (2015), consumers primarily seek lower levels of anxiety and greater support during the purchase process. Nian et al. (2019) highlight that the new model requires the dealer to take a proactive role, emphasizing the importance of clear and effective communication. Wangchuk et al. (2024) add that a positive attitude toward electric vehicles significantly influences consumer receptivity, reinforcing the need for salespeople to possess well-developed interpersonal skills to build trusting relationships. This finding reinforces the importance of developing soft skills not only for selling but also for building long-term customer relationships.

Finally, the increasing digitization of the marketplace is an aspect salespeople must skillfully manage. As consumers increasingly use digital platforms to inform themselves and compare options before making a purchase, marketers will need to adapt to this trend. Li et al. (2023) highlight the importance of online interactions, including the perceived convenience and aesthetics of automotive companies’ digital platforms and the appeal of social media, as factors contributing to functional experience value (FEV). The research highlights the need for automotive companies to develop robust and engaging digital platforms that provide comprehensive information and a positive user experience to maximize their impact on purchase decisions.

There is ample evidence that challenges and opportunities exist for EV marketers. They must evolve to address cost and range concerns, provide information on charging infrastructure, and master social competencies that facilitate the consumer experience. A deep understanding of the product and the ability to build a consultative relationship with the customer are key to success in electric vehicle marketing today and in the future.

## 7. Skills and Competency Development Strategy for Electric Vehicle Marketers

We know that the automotive industry is undergoing a profound transformation with the irruption of electric vehicles (EVs). This transition, driven by the need for sustainable mobility (Adnan et al., 2017; Cruz-Jesus et al., 2023) and the growing concern about climate change (Pamidimukkala et al., 2023), redefines not only the product itself but also the market dynamics and the seller’s role (Lam & Mercure, 2020). Despite the potential of EVs for transport decarbonization and for meeting the Sustainable Development Goals (SDGs) (Omahne et al., 2021), their adoption faces significant barriers, including high upfront costs (Abas & Tan, 2024; Anosike et al., 2023), limited autonomy (Barter et al., 2015; Bjerkan et al., 2016), and a lack of charging infrastructure (Mpoi et al., 2023; Munshi et al., 2022).

This complexity demands a more specialized salesperson profile (Anderson et al., 2022; Genzlinger et al., 2020). The traditional sales approach, focused on the transaction, is no longer sufficient. Consumers arrive informed, comparing options and looking for comprehensive solutions (Bacher & Manowicz, 2020; Singh et al., 2015). The salesperson must evolve into a consultative role (Paesbrugghe et al., 2020), understanding individual mobility needs and offering customized solutions (Zarazua de Rubens et al., 2020). These areas of empowerment will not only improve sales efficiency but will also contribute to sustainable EV market growth and customer satisfaction. The buying experience, mediated by the salesperson-customer interaction, can be decisive in convincing consumers (Matthews et al., 2017; Tromaras et al., 2017).

### 7.1. Skills to Develop and Their Application in the Marketing of Electric Cars

Based on findings from various research, several key competencies that electric car marketers need to develop are identified. These findings are grouped into different categories.

#### 7.1.1. Analytical Thinking and Scrupulousness for Objections Management

The main barrier to EV adoption is their high initial cost and limited autonomy (Abas & Tan, 2024; Anosike et al., 2023; Barter et al., 2015; Bjerkan et al., 2016). The development of analytical thinking (Das et al., 2018; Hsiao, 2012) and scrupulousness (Lilford et al., 2014; Mostafa & Kasamani, 2022) enables the marketer to analyze individual customer needs, presenting detailed comparisons between initial cost and long-term savings, considering factors such as energy costs and reduced maintenance. Scrupulousness will enable an honest and transparent presentation of information, build trust, and minimize uncertainty about long-term value by effectively responding to customer objections about price and autonomy.

#### 7.1.2. Extroversion and Effective Communication Skills for Proactivity

Lack of charging infrastructure is a significant concern (Mpoi et al., 2023; Munshi et al., 2022). Extroversion (Lilford et al., 2014; Mostafa & Kasamani, 2022) and effective communication skills (Omar, 2017, 2018) enable the salesperson to proactively address the charging concern by providing detailed information on charging station locations, charging plans, and customized solutions for each customer. Effective communication will enable this information to be conveyed clearly and understandably, dispelling doubts and building trust. Extroversion facilitates proactive interaction with the customer and anticipates their needs.

#### 7.1.3. Empathy and Active Listening Skills for Customer Connection

Consumers seek complete information and support during the buying process (Li et al., 2023; Lim et al., 2015; Nian et al., 2019; Wangchuk et al., 2024). Empathy (Agnihotri et al., 2012, 2016) and active listening skills (Omar, 2017, 2018) are essential to understand individual needs and concerns. These skills help build a trusting relationship with the customer, creating a positive experience that influences the purchase decision.

#### 7.1.4. Adaptability and Continuous Learning for Innovation: Adaptability and Continuous Learning for Expert Advice

The EV market is dynamic and requires constant adaptation to changes, and a deep understanding of EV technical characteristics, including autonomy, charging times, battery types, propulsion systems, and charging technologies, is critical (Barter et al., 2015; Bjerkan et al., 2016). Adaptability and continuous learning (Phongkhajeerathibha et al., 2023; Rantala et al., 2020) are fundamental to staying up-to-date on new technologies, regulations, and market trends. This technical knowledge enables the salesperson to accurately answer customer questions, allay technology-related fears, and provide expert advice, building trust and facilitating purchase decisions.

#### 7.1.5. Mindfulness and Courtesy for Customer Experience

**Figure 6:**
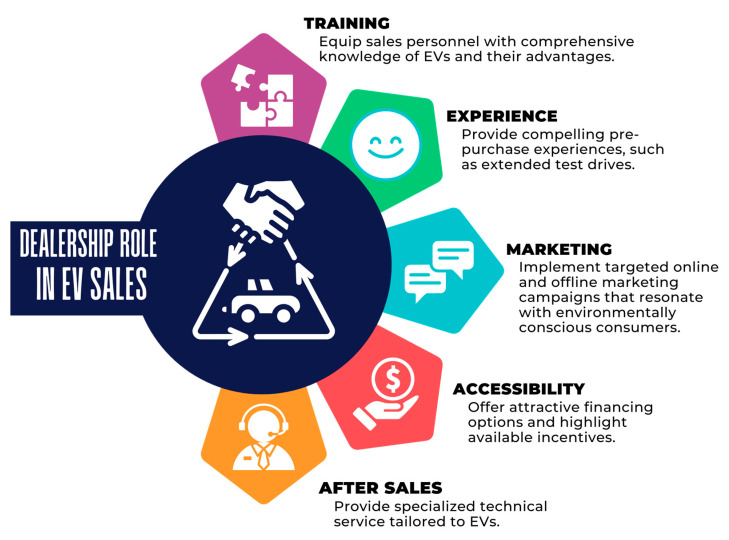
Competency development strategy for EV salespeople.

Mindfulness (Charoensukmongkol & Pandey, 2021) enables the salesperson to be present in the interaction, offer personalized service, and be attentive to the customer’s needs, thereby generating a positive experience that influences the purchase decision. Politeness (Das et al., 2018; Hsiao, 2012) ensures a positive and professional interaction across all communication channels, contributing to an exceptional customer experience. Consequently, based on the findings of the present research, a competency development strategy for EV salespeople has been developed and shown in Figure 6.

### 7.2. Discussion on the Dealership Role in EV Sales

Building on the study’s focus on social–cognitive skills in sales and on the competency development strategy for EV salespeople shown in Figure 6, this section examines how dealerships encourage EV adoption through practices that activate key psychological mechanisms, such as empathy, adaptability, trust-building, and perspective-taking.

#### 7.2.1. Training

Dealerships need to shift their training approach from solely transactional product and price scripts to a blended curriculum. This finding should combine EV technical knowledge, such as range, charging, battery life, and total cost of ownership, with social–cognitive skills like empathy, active listening, and consultative questioning. The present study emphasizes that, given the technological complexity of EVs and customers’ concerns about range and charging, salespeople must be able to provide honest, expert explanations and personalized cost comparisons. Training programs should therefore include: technical modules covering charging behavior, home versus public charging, and interoperability; role-plays to build empathy and perspective-taking; objection-handling with analytical total cost of ownership tools; and short, practical drives to help reduce experiential anxiety. Continuous investment in upskilling and refresher modules is crucial, given the rapid evolution of the EV market and infrastructure. This training supports psychological models of social–cognitive functioning because skills such as empathy, adaptability, active listening, and mindful perspective-taking improve a salesperson’s capacity to understand customer needs, which aligns with studies on mentalizing and interpersonal skills in sales performance (Agnihotri et al., 2012; Omar, 2017, 2018).

#### 7.2.2. Experience (Customer and Salesperson Experience)

The study emphasizes that hands-on experience with EVs, such as test drives, demonstrator units, and short experiential events, significantly boosts purchase consideration. Additionally, a knowledgeable and attentive salesperson enhances customer trust and reduces anxiety. Dealerships should create in-store experiences that showcase charging connectors, offer simulated route-range calculations, and include brief test drives focused on everyday use. To improve salesperson expertise, rotate staff through technical workshops and visits to charging stations, enabling them to speak from personal experience rather than just technical specifications. Lastly, design the showroom to encourage low-pressure exploration, such as a charging demo area and clear comparison charts, to foster positive and engaging experiential learning for customers. This focus on experiential encounters is reinforced by psychological theories that demonstrate adaptability, trust, emotional attunement, and perceived authenticity develop through direct interpersonal interactions, which are supported by empathy and mindfulness as shown in sales research (Charoensukmongkol & Pandey, 2021; Omar, 2018).

#### 7.2.3. Marketing

The review suggests that marketing should shift from just listing features to creating narratives that lower perceived risks and highlight long-term benefits, convenience (like charging solutions), and environmental identity tailored to different customer segments. Dealers ought to coordinate their digital and in-store messages by using clear websites and social media posts that detail local charging coverage and total cost of ownership (TCO) calculators, complemented by in-store materials such as infographics and short videos that reflect the online story. Tailoring messages for specific segments is crucial, for example, cost-saver packages for budget-conscious buyers, tech/comfort narratives for early adopters, and convenience bundles (including home charger and installation services) for mainstream consumers. Incorporating social proof, such as local owner testimonials and simple comparisons, can help build trust and address resale and range anxiety, as documented in existing research. Incorporating these social–cognitive components into marketing demonstrates that successful persuasion in EV contexts depends not only on providing information but also on the salesperson’s skill in understanding customer perspectives and establishing trust through transparent and empathetic communication (Mostafa & Kasamani, 2022; Omar, 2017).

#### 7.2.4. Accessibility (Charging, Purchasing Channels, & Physical Access)

The study repeatedly emphasizes that accessible charging infrastructure and convenient access are key to encouraging adoption; therefore, dealerships should serve as connectors to accessibility rather than just sales points. This finding involves providing bundled solutions such as home charger procurement and installation, information from preferred public-charging partners, clear maps of local chargers, and financing or leasing options that include expected charging costs. Dealerships should also diversify purchase methods, adopting hybrid online-offline processes in which customers can research and begin paperwork online, while experiential steps like test drives and final consultations take place in the showroom. Lastly, ensure showrooms are physically and informationally accessible, with staff available for brief questions-and-answers sessions, simple charging setup guides, and multilingual or jargon-free materials for underserved communities. From a social–cognitive perspective, enhancing adaptability and accessibility not only boosts perceived support but also lessens uncertainty. This fact aligns with research showing that customer reassurance depends on the salesperson’s ability to provide empathetic guidance and perspective-taking (Lim et al., 2015; Nian et al., 2019).

#### 7.2.5. After Sales (Service, Warranty, Resale Support)

After-sales support is highlighted in this review as essential for reducing concerns about EV resale value and reliability. Dealerships should provide transparent warranties for batteries, clearly outline maintenance schedules, and, when possible, offer resale-value guarantees or certified pre-owned options to ease concerns about future risks. Service teams need training in EV diagnostics and should communicate the lower maintenance costs compared to internal-combustion-engine vehicles to emphasize routine savings. Regular after-sales interactions such as battery health reports, charging advice, and software update summaries, along with quick responses to customer inquiries, will build trust and encourage positive word-of-mouth, especially in markets where social proof influences adoption. This focus on after-sales service aligns with psychological research indicating that long-term trust and perceived reliability in relationships are built through consistent empathic communication and continuous social–cognitive interaction between customers and salespeople (Charoensukmongkol & Pandey, 2021; Omar, 2018).

## 8. Conclusions: Industry Implications

The implementation of the EV salesperson competency development strategy described in this research will have a profound and multifaceted impact on the industry. It is not just an increase in sales, but a transformation in how this technology is perceived and adopted. First, the strategy will directly address key consumer concerns, such as high initial cost, limited autonomy, and availability of charging infrastructure. By equipping marketers with the skills to analyze individual customer needs, offer customized solutions, and allay fears with accurate and transparent information, greater confidence in electric vehicles will be generated. This confidence will translate into a significant acceleration in adoption rates, driving market growth beyond current projections.

In addition to the direct impact on sales, the strategy will substantially improve the customer experience. Personalized attention, effective communication, and expert advice from highly trained salespeople will create a positive, memorable shopping experience. This positive experience will not only increase customer satisfaction but will also foster brand loyalty and generate positive recommendations through word of mouth and social media. This multiplier effect is significant for the industry’s long-term success, as customer trust and satisfaction are key drivers of the widespread adoption of electric vehicles.

Beyond its effect on individual consumers, the strategy will help improve the overall image of the EV industry. The professionalization of the sales force, with its technical knowledge and communication skills, will help dispel the myths and negative perceptions that still persist around this technology. A more positive, trustworthy image will attract more consumers, accelerating the transition to electric mobility and consolidating its position as a viable and attractive alternative. This improved public perception will also attract additional investment to the industry, driving innovation and the development of new technologies.

This finding underscores the importance of recognizing that investment in sales training, while an initial cost, will generate significant returns in the long term. A highly trained sales force will be more efficient, reducing the costs associated with after-sales service and complaint management. In addition, lower staff turnover and higher customer satisfaction will contribute to the sector’s stability and profitability.

In summary, the dealership’s role in EV sales highlights that successful adoption requires more than just technical knowledge and product availability. It depends on a relational strategy rooted in social–cognitive skills, including empathy, adaptability, perspective-taking, and trust-building communication. Enhancing training, providing experiential opportunities, refining customer-focused marketing, improving access to charging and purchase options, and offering dependable after-sales support all hinge on these psychological competencies. As this study indicates, although some electric vehicles are sold through direct-to-consumer channels without salespeople, these skills are crucial for reducing customer uncertainty and creating positive EV purchase experiences, underscoring that the human element remains vital even amid advanced technological shifts in mobility.

## Data Availability

Data is available from the authors upon reasonable request.

## Appendix A

### Appendix A.1

Table A1 presents the articles analyzed from the search for key competencies and skills in salespeople across various industries and sectors.

**Table A1:** Articles analyzed from the search for key competencies and skills in salespeople from different industries and sectors.

Authors	Year	Author (Year)	Title of Publication	Findings
Agnihotri, R., Krush, M., & Singh, R. K	2012	Agnihotri et al. (2012)	Understanding the mechanism linking interpersonal traits to pro-social behaviors among salespeople: Lessons from India.	This study in India reveals that empathy and guilt proneness are key emotional traits for sales success. Empathy drives internal collaboration, while guilt proneness drives responsible actions with customers, improving satisfaction and retention. Although empathy did not directly affect customer performance, both traits contribute to overall sales performance, with internal collaboration being a more influential factor. This finding underscores the importance of emotional intelligence in sales, especially in emerging markets.
Agnihotri, R., Vieira, V. A., Senra, K. B., & Gabler, C. B.	2016	Agnihotri et al. (2016)	Examining the impact of salesperson interpersonal mentalizing skills on performance: the role of attachment anxiety and subjective happiness.	This study in Brazil demonstrates that salesperson interpersonal mentalizing skills, positively influenced by happiness and negatively influenced by attachment anxiety, predict sales performance in sectors such as telecommunications, banking, and retail. Improving emotional well-being and interpersonal skills enhances sales team effectiveness.
Ahmad, S. Z., Sah, B. M., & Kitchen, P. J.	2010	Ahmad et al. (2010)	The relationship between sales skills and salesperson performance, and the impact of organizational commitment as a moderator: An empirical study in a Malaysian telecommunications company.	In the telecommunications industry, interpersonal skills are central to sales success, unlike other competencies such as technical or marketing skills. Recruitment and training strategies should prioritize the development of these skills to improve overall performance.
Al-Assaf, K. T., & al Muala, A. M.	2023	Al-Assaf and Al Muala (2023)	The Impact of Salesperson Skills on the Purchasing Decision in Electronics Showroom During the Coronavirus (COVID-19) Pandemic in Zarqa City.	The pandemic accelerated the need for salespeople to enhance their digital skills to interact with customers across various platforms. This adaptability to new technologies improves immediate performance and cultivates long-term customer relationships.
Charoensukmongkol, P., & Pandey, A.	2021	Charoensukmongkol and Pandey (2021)	Trait mindfulness and cross-cultural sales performance: The role of perceived cultural distance.	In international sales, mindfulness, sales planning, cultural awareness, adaptability, and effective communication are vital. Mindfulness improves planning, allowing strategies to be adapted to the expectations of foreign customers, especially in culturally diverse contexts.
D’Ament, G., Nayeem, T., & Saliba, A. J.	2024	D’Ament et al. (2024)	Personality, mood, or emotion? Influence of customer trait and state during the cellar door experience on sales and word-of-mouth intention: A Bayesian approach.	Wine sales personnel must possess general knowledge and adapt their communication to the customer’s level of experience, actively listening to the customer’s needs. Ongoing training and creating a welcoming environment are essential investments to encourage spending and positive recommendations.
Das, M. R., Pathak, P., & Singh, S.	2018	Das et al. (2018)	Competency identification of salespersons through behavioral event interviews: Evidence from the oil industry.	In the Indian oil industry, politeness, honesty, service orientation, and punctuality are key competencies that predict sales performance. These skills build trust, strengthen relationships, and increase customer satisfaction, driving sales growth.
Hsiao, J.-M.	2012	Hsiao (2012)	Exploring the effects of salesperson competencies on performance: Case study of a Ford car dealer in Taiwan.	Analytical thinking improves salesperson performance, while achievement orientation has a negative impact. Interpersonal skills, leadership, and flexibility are also important. Developing analytical thinking and reassessing achievement orientation are decisive for improving performance.
Imran, F., & Kantola, J.	2018	Imran and Kantola (2018)	A co-evolute approach to analyze the competencies of sales personnel of banking sector of Pakistan.	This research analyzes the competencies of sales personnel in the Pakistani banking sector, a highly competitive sector. Using the Evolute system and the Astroid 1.0 tool, the competencies and creative tension of employees were assessed. The study developed a practical framework to improve sales staff skills, contributing to better human resource management practices in the Pakistani banking sector. It seeks to develop the competencies necessary for success in a competitive environment.
Joya, N., Aline, H., Katia, I., Hala, S., Marwan, A., Rony, Z. M., Chadia, H., & Pascale, S.	2023	Namnoum et al. (2023)	Assessing and validating the specialized competency framework for pharmacists in sales and marketing (SCF-PSM): a cross-sectional analysis in Lebanon.	A competency framework for pharmacists in sales and marketing in Lebanon highlights the need to improve skills in pharmaceutical knowledge, professional communication, emergency preparedness, and operational management. Deficiencies are identified in negotiation, data processing, information technology, self-management, and professional ethics.
Kakeesh, D. F., Al-Weshah, G. A., & Dababneh, R. B.	2021	Kakeesh et al. (2021)	Leveraging market share through selling skills: The mediating role of adaptive selling behavior.	In pharmaceutical sales, nonverbal immediacy, empathy, experience, and subjective knowledge are essential competencies that significantly influence market share and performance. Continuous training in these areas is vital to improve sales effectiveness and customer relationships.
Kazén, M., Kuhl, J., Boermans, S., & Koole, S. L.	2013	Kazén et al. (2013)	Excelling at selling: The charming personality style predicts occupational activities, sales performance, and persuasive competence.	In a German insurance company, a charming personality predicted branch managers’ sales performance over two years. Explicit power motivation (desire to influence others) partially mediated this relationship, indicating that charming people are motivated to exert influence, thus improving their sales.
Kimura, T., Bande, B., & Fernández-Ferrín, P.	2019	Kimura et al. (2019)	The roles of political skill and intrinsic motivation in performance prediction of adaptive selling.	Political skill and intrinsic motivation enhance the effectiveness of adaptive selling. Political skill facilitates internal networking, while intrinsic motivation drives adaptation, improving sales performance.
Lilford, N., Vigar-Ellis, D., & Nel, D.	2014	Lilford et al. (2014)	Big Five personality traits and financial salesperson performance: An application of Chernoff faces	Conscientiousness is a key personality trait that influences sales performance, yet these traits have limited explanatory power in predicting sales success.
Mostafa, R. B., & Kasamani, T.	2022	Mostafa and Kasamani (2022)	The Effect of Salespeople Skills on Selling Behaviors: The Moderating Role of Social Media.	Selling skills, technical skills, active empathic listening, and emotional intelligence positively influence adaptive and cross-selling. The use of social media enhances the effectiveness of these skills.
Omar, N.	2017	Omar (2017)	Methods of discovering effective listening skills in direct selling actual conversations.	Communication competence is key in direct selling. Punctuality, a friendly attitude, attention to similarities, active listening, nonverbal communication, a sense of humor, relevant information, and timely completion of sessions, keeping calm, are key elements.
Omar, N.	2018	Omar (2018)	The relationship components of communication competence in the direct selling process in Malaysia.	Effective listening skills are important for building relationships, improving communication, and achieving sales goals in direct selling. A study combining interviews, observations, and surveys identified key competencies, including concentration, minimizing interruptions, and coordinating nonverbal cues.
Phongkhajeerathibha, E., Mohan, K. P., & Tuntivivat, S.	2023	Phongkhajeerathibha et al. (2023)	Developing Instrument for Micro-Skills of Selling for Sales Professionals in the Thai Petrochemical Industry.	A scale was developed to measure micro-skills of selling (listening, questioning, and presentation) in the petrochemical industry. Using mixed methods, its high reliability and validity were demonstrated, highlighting the role of these skills in sales performance and customer relationships.
Ping, Z. Y., Yun, L., Ying, Y. X., & Ran, T.	2014	Ping et al. (2014)	Competences of frontline sales staff: A case study on a medicines sales company.	Nine key competencies were identified for medicine sales staff: perseverance, self-motivation, and internal rectification. Successful salespeople show greater self-reflection and take responsibility for failures, unlike ordinary staff who attribute failures to external factors.
Rantala, J., Yllikäinen, M., & Holopainen, T.	2020	Rantala et al. (2020)	Competences in Expert Sales	In the financial sector, especially in expert sales, understanding of the customer experience, effective communication, and in-depth product knowledge are emphasized. Personalized value creation, adaptability, collaboration, analytical skills, and networking are critical for success.
Retnawati, B. B., & Nuryakin, N.	2016	Retnawati and Nuryakin (2016)	Developing salesperson performance: The role of customer encountering competence portfolio, relational capital and service excellent customer heterogeneity.	Relational capital and service excellence significantly improve sales performance in the pharmaceutical industry. Strong relationships based on trust and open communication, coupled with high-quality personalized service, increase customer loyalty and sales performance.
Sitser, T., van der Linden, D., & Born, M. P.	2013	Sitser et al. (2013)	Predicting Sales Performance Criteria With Personality Measures: The Use of the General Factor of Personality, the Big Five and Narrow Traits.	Conscientiousness and openness are significant personality traits for sales performance. Proactivity and attention to detail enhance specific tasks, while thoughtfulness supports customer relationship management. Social boldness, however, negatively affects performance.
Sypniewska, B. A.	2013	Sypniewska (2013)	Examination of the individual competencies that differentiate results in direct sales.	In multilevel marketing (MLM), leadership, team management, knowledge, self-motivation, and customer orientation significantly influence sales performance. Leadership and team management are important for experienced salespeople, while self-motivation differentiates the best.
Tornillo, J. E., Pascal, G., Moguerza, J. M., & Redchuk, A.	2019	Tornillo et al. (2019)	Personality Traits and Business Intelligence: A Model to Improve Direct Selling Systems.	In direct selling, the DISC test highlights long-term reliability, self-motivation, proactivity, and decision-making under pressure, as well as agreeableness and persuasiveness. Profiles with high Influence (I) excel in interpersonal relationships, while CI and DS profiles perform better in sales.
Waheed, A., Yang, J., & Webber, J.	2017	Waheed et al. (2017)	The effect of personality traits on sales performance: An empirical investigation to test the five-factor model (FFM) in Pakistan.	The Five-Factor Model (FFM) shows that extraversion is the most significant predictor of sales performance, followed by agreeableness. Conscientiousness also contributes positively, while emotional stability and openness to experience have a weaker correlation.
Wihler, A., Meurs, J. A., Momm, T. D., John, J., & Blickle, G.	2017	Wihler et al. (2017)	Conscientiousness, extraversion, and field sales performance: Combining narrow personality, social skill, emotional stability, and nonlinearity.	Conscientiousness and extraversion enhance sales performance through Disciplined Achievement Motivation (DAM) and Stable Social Potentiality (SSP). A high level of DAM can boost performance initially, but excess can lead to perfectionism. SSP moderates this relationship, allowing adaptation to stress and rejection. High levels of DAM and SSP achieve better results.

### Appendix A.2

Table A2 presents the articles analyzed from the search for the main factors influencing the purchase and/or adoption of electric cars.

**Table A2:** Articles analyzed from the search for the main factors influencing the purchase and/or adoption of electric cars.

Authors	Year	Author (Year)	Title	Main Findings
Abas, P. E., & Tan, B.	2024	Abas and Tan (2024)	Modeling the Impact of Different Policies on Electric Vehicle Adoption: An Investigative Study	High upfront costs, limited range, and poor infrastructure slow electric vehicle adoption, requiring supportive policies to incentivize purchase and achieve market targets.
Anosike, A., Loomes, H., Udokporo, C. K., & Garza-Reyes, J. A.	2023	Anosike et al. (2023)	Exploring the challenges of electric vehicle adoption in final mile parcel delivery.	High initial prices, uncertainty about residual values, and the lack of a second-hand market are barriers to electric vehicle adoption. Fleets must consider the total cost of ownership.
Barter, G. E., Tamor, M. A., Manley, D. K., & West, T. H.	2015	Barter et al. (2015)	Implications of modeling range and infrastructure barriers to adoption of battery electric vehicles.	Adoption of battery electric vehicles (BEVs) is limited (5% in 2050) due to price, range, charging infrastructure, and charging times, as modeled using GPS travel data.
Bjerkan, K. Y., Nørbech, T. E., & Nordtømme, M. E.	2016	Bjerkan et al. (2016)	Incentives for promoting Battery Electric Vehicle (BEV) adoption in Norway.	Fiscal incentives for purchase, such as tax exemptions, play a key role. Other incentives, such as access to bus lanes and toll exemptions, are decisive for specific groups.
Bjorge, N. M., Hjelkrem, O. A., & Babri, S.	2022	Bjørge et al. (2022)	Characterisation of Norwegian Battery Electric Vehicle Owners by Level of Adoption	Early EV adopters prioritized environmental friendliness, while the early majority focused on low operating costs. Economic incentives are central for mass adoption.
Canepa, K., Hardman, S., & Tal, G.	2019	Canepa et al. (2019)	An early look at plug-in electric vehicle adoption in disadvantaged communities in California.	EV buyers tend to have higher incomes and higher levels of education. Financial incentives, such as rebates and access to VAO lanes, drive sales. Charging infrastructure, especially at home, is a component of adoption.
Chinen, K., Matsumoto, M., Liu, C., Tong, P., Han, Y. S., & Endo, H.	2023	Chinen et al. (2023)	Expanding electric-vehicle adoption beyond the national border: Insights for developing marketing policies for global electric-vehicle manufacturers.	Environmental awareness influences electric-vehicle purchase through perceptions of quality and value. Animosity toward China and ethnocentrism negatively affect purchase intention.
Rubens, G. Z., Noel, L., & Sovacool, B. K.	2018	Zarazua de Rubens et al. (2018)	Dismissive and deceptive car dealerships create barriers to electric vehicle adoption at the point of sale.	Car dealers dismiss electric vehicles (EVs), misinform buyers, and steer them towards gasoline- or diesel-powered vehicles. The salesperson’s orientation, knowledge of EVs, and willingness to sell EVs influence the likelihood of purchase.
Dimanchev, E., Qorbani, D., & Korpås, M.	2022	Dimanchev et al. (2022)	Electric Vehicle Adoption Dynamics on the Road to Deep Decarbonization.	Mass adoption of electric vehicles requires sustained supportive policies that overcome barriers such as range, charging speed, and consumer awareness, as the case of Norway demonstrates.
Dua, R., Hardman, S., Bhatt, Y., & Suneja, D.	2021	Dua et al. (2021)	Enablers and disablers to plug-in electric vehicle adoption in India: Insights from a survey of experts.	High initial prices, lack of charging infrastructure, and insufficient support policies are the main barriers to electric vehicle adoption in India. Subsidies and mandate/feebates policies can drive demand.
Dua, R., White, K., & Lindland, R.	2019	Dua et al. (2019)	Understanding potential for battery electric vehicle adoption using large-scale consumer profile data.	BEV buyers are looking for style, increased range, all-wheel drive, and competitive pricing. Success depends on meeting the needs of the most demanding segment that prioritizes these features.
Forsythe, C. R., Gillingham, K. T., Michalek Jeremy J., and Whitefoot, K. S.	2023	Forsythe et al. (2023)	Technology advancement is driving electric vehicle adoption	Technology improvements, especially in range and price, are driving electric vehicle adoption, with consumer valuation projected to equal or exceed that of gasoline vehicles by 2030.
Goswami, R.	2022	Goswami (2022)	Factors influencing the adoption of electric vehicles in India: an empirical analysis.	Consumers prioritize price, performance (range and charging time), and charging infrastructure when considering an electric vehicle purchase. Environmental and social awareness also play a role.
Graham, J. D., & Brungard, E.	2021	Graham and Brungard (2021)	Consumer Adoption of Plug-In Electric Vehicles in Selected Countries	Consumers seek sporty performance, low operating costs, and convenience in EVs, but high initial price, limited range, and slow charging are barriers to mass adoption. Incentive policies and disincentives to conventional vehicles drive commercialization.
Hathaway, Z., Polis, H., Loomis, J., Boroski, J., Milano, A., & Ouyang, J.	2021	Hathaway et al. (2021)	A utility roadmap for expanding customer adoption of electric vehicles.	Consumers are primarily looking for lower operating and environmental costs in EVs. Price and vehicle range remain significant barriers, especially for low-income and minority communities. Test-drive events increase purchase interest.
Higueras-Castillo, E., Guillen, A., & Herrera Luis-Javier and Liebana-Cabanillas, F.	2021	Higueras-Castillo et al. (2021)	Adoption of electric vehicles: Which factors are really important?	Autonomy, incentives, and reliability are the main predictors of EV purchase intention. Consumers prioritize these technological and contextual factors.
Hemalatha, J., Panboli, S., & Aravindh Kumaran, L.	2024	Hemalatha et al. (2025)	Electric vehicle adoption toward sustainable transportation solution: key drivers and implications.	Consumers value environmental, social, technological orientation, and monetary benefits when considering the purchase of an electric vehicle. Positive attitude toward EVs mediates purchase intention.
Jang, S., & Choi, J. Y.	2021	Jang and Choi (2021)	Which consumer attributes will act crucial roles for the fast market adoption of electric vehicles: Estimation on the asymmetrical and heterogeneous consumer preferences on the EVs.	Consumers prioritize purchase price, fuel efficiency, range, and fast charging when buying an EV. They exhibit loss aversion when comparing prices with those of their previous vehicle.
Kennedy, D., & Philbin, S. P.	2019	Kennedy and Philbin (2019)	Techno-economic analysis of the adoption of electric vehicles.	Electric vehicles are gaining popularity, but purchase decisions are driven by perceptions of low cost and environmentalism, not always aligned with life-cycle analysis.
Khusanboev, I., Yodgorov, I., & Karimov, B.	2023	Khusanboev et al. (2023)	Advancing Electric Vehicle Adoption: Insights from Predictive Analytics and Market Trends in Sustainable Transportation	Consumers prioritize predictable range, energy efficiency, and competitive selling price when considering the purchase of an electric vehicle.
Li, W., Wang, M., Cheng, X., & Long, R.	2023	Li et al. (2023)	The impact of interaction on the adoption of electric vehicles: Mediating role of experience value.	In electric car marketing, customers value informative interactions, hands-on experiences, and knowledgeable and enthusiastic salespeople. Digital platforms and apps play an important role in the modern shopping experience.
Lim, M. K., Mak, H.-Y., & Rong, Y.	2015	Lim et al. (2015)	Toward mass adoption of electric vehicles: Impact of the range and resale anxieties.	Sales strategies should mitigate range anxiety and resale anxiety by offering battery leasing, guaranteeing charging infrastructure, and highlighting vehicle reliability.
Moeletsi, M. E.	2021	Moeletsi (2021)	Socio-economic barriers to adoption of electric vehicles in South Africa: Case study of the Gauteng province.	The purchase price and battery costs are the main barriers to the adoption of electric vehicles in South Africa. Consumers value long-term savings and extended warranties.
Mpoi, G., Milioti, C., & Mitropoulos, L.	2023	Mpoi et al. (2023)	Factors and incentives that affect electric vehicle adoption in Greece.	Government incentives, charging infrastructure, and environmental awareness are key. Buyers prioritize low cost, autonomy, and a charging network. Sellers should emphasize these aspects.
Munshi, T., Dhar, S., & Painuly, J.	2022	Munshi et al. (2022)	Understanding barriers to electric vehicle adoption for personal mobility: A case study of middle income in-service residents in Hyderabad city, India.	High upfront costs, lack of charging infrastructure, and concerns about operating costs are key barriers. Financial incentives and policies that improve convenience and reduce uncertainty help adoption.
Murugan, M., Marisamynathan, S., & Nair, P.	2023	Murugan et al. (2023)	Investigating the Barriers for Electric Vehicle Adoption Using Analytical Hierarchy Process Approach.	High initial price, limited range, unreliable battery technology, and limited availability of charging stations are the main barriers to EV adoption in India. Unknown resale value and low availability of local technicians are secondary barriers.
Mustafa, S., Shi, Y., Adan, D. E., Luo, W., and al Humdan, E.	2024	Mustafa et al. (2024)	Role of environmental awareness & self-identification expressiveness in electric-vehicle adoption.	Perceived value (benefits minus sacrifices) and perceived usefulness are the main drivers of EV purchase. Environmental awareness is a factor, but cost is a significant barrier.
Nazari, F., Noruzoliaee, M., & Mohammadian, A.	2024	Nazari et al. (2024)	Electric Vehicle Adoption Behavior and Vehicle Transaction Decision: Estimating an Integrated Choice Model with Latent Variables on a Retrospective Vehicle Survey.	Social influence drives EV adoption, especially among those sensitive to trends and advertising. A high purchase price, especially for plug-ins, discourages ownership and favors leasing. The availability of charging infrastructure in suburban areas also plays a role.
Nian, V., Hari, M. P., & Yuan, J.	2019	Nian et al. (2019)	A new business model for encouraging the adoption of electric vehicles in the absence of policy support.	The new model calls for the dealer to take on financial and energy management functions, requiring skills in financing, electricity trading, charging logistics, and cross-sector collaboration, transforming into an electric mobility service provider.
Nimesh, V., Manoj, B. S., Bhaduri, E., Mahendra Reddy, V., & Kishore Goswami, A.	2024	Nimesh et al. (2024)	Estimating personal electric vehicle demand and its adoption timeframe: A study on consumer perception in Indian metropolitan cities.	Indian consumers value price, range, and charging time when considering electric vehicles. Poor infrastructure and negative perception hinder purchase.
Pajic, N., Jurisevic, N., & Despotovic, M.	2024	Pajic et al. (2024)	Adoption of Electric Vehicles in the Republic of Serbia	Studies show that financial incentives drive the adoption of electric vehicles. Consumers prioritize range, price, and charging infrastructure. Salesperson experience and available information influence the purchase decision.
Paradies, G. L., Usmani, O. A., Lamboo, S., & van den Brink, R. W.	2023	Paradies et al. (2023)	Falling short in 2030: Simulating battery-electric vehicle adoption behavior in the Netherlands.	Consumers seek competitive prices, greater autonomy, and consider social factors such as the influence of others. Routine purchases and the desire to “fit in” are significant barriers to the adoption of electric vehicles.
Plananska, J., & Gamma, K.	2022	Plananska and Gamma (2022)	Product bundling for accelerating electric vehicle adoption: A mixed-method empirical analysis of Swiss customers.	Electric vehicle (EV) bundling with charging services increases willingness to purchase, especially among customers with little prior knowledge. Three customer segments with distinct package preferences are identified: technology-oriented, convenience-oriented, and low adoption probability.
Priessner, A., Sposato, R., & Hampl, N.	2018	Priessner et al. (2018)	Predictors of electric vehicle adoption: An analysis of potential electric vehicle drivers in Austria.	Psychological factors (pro-environmental and technological attitudes) better predict electric vehicle adoption than sociodemographic factors. Four segments of potential buyers with different incentive preferences are identified. Marketing should focus on attitudes, not just demographics.
Ramadan, M., & Othman, M.	2023	Ramadan and Othman (2024)	Psychological antecedents of electric vehicle adoption in the West Bank.	Consumers prioritize incentives, product knowledge (range, charging, etc.), and environmental concerns when considering EV purchase. Perceived behavioral control was not significantly influenced.
Roberson, L. A., & Helveston, J. P.	2020	Roberson and Helveston (2020)	Electric vehicle adoption: Can short experiences lead to big change?	The study shows that a brief experience of driving an electric vehicle significantly increases purchase consideration, even with low prior knowledge. Consumers value direct experience, rather than technical knowledge.
Salari, N.	2022	Salari (2022)	Electric vehicles adoption behavior: Synthesising the technology readiness index with environmentalism values and instrumental attributes.	Consumers value charging convenience, driving range, and fuel cost savings. Technology readiness and innovation are key factors, while the desire for unique products is less important. Education about maintenance costs and range is highlighted.
Shankar, A., & Kumari, P.	2019	Shankar and Kumari (2019)	Exploring the enablers and inhibitors of electric vehicle adoption intention from sellers’ perspective in India: A view of the dual-factor model.	This study investigates why electric vehicle (EV) sales are low despite their benefits. It analyzes what motivates and prevents sellers from promoting EVs. They found that positive attitudes, social norms, and perceived control drive marketers’ intention, while resistance to change, fear of regret, and preference for traditional cars hold them back.
Shareeda, A., Al-Hashimi, M., & Hamdan, A.	2021	Shareeda et al. (2021)	Smart cities and electric vehicles adoption in Bahrain	Bahraini consumers prioritize electric vehicle awareness and purchasing power. Vehicle range is less important. Charging infrastructure and government support are required.
Sinha, N., Jain, V., & Sehrawat, R.	2024	Sinha et al. (2024)	Mapping the Factors Influencing the Adoption of Electric Vehicles: A Literature Synthesis	Consumers seek real environmental benefits and perceived value. Government policies, charging infrastructure, and after-sales services are important. The importance of direct experience with electric vehicles is highlighted.
Wang, N., & Liu, Y.	2015	Wang and Liu (2015)	City Readiness System Assessment of Electric Vehicle Adoption in China.	The study highlights the importance of purchase subsidies and adequate charging infrastructure in driving electric vehicle adoption. “Range anxiety” and lack of consumer awareness are obstacles.
Wangchuk, S., Mahajan, P., Abhimanyu, M. A., & Chaudhary, R.	2024	Wangchuk et al. (2024)	Assessment of North Delhi People’s Electric Vehicle Purchase Intent through Consumer Behavior and Technology Adoption Theory.	Positive attitude towards electric vehicles influences purchase intent, but there is no direct relationship with their attributes. A better understanding of consumer perceptions is required.
Wei, W., Cao, M., Jiang, Q., Ou, S.-J., & Zou, H.	2020	Wei et al. (2020)	What influences Chinese consumers’ adoption of battery electric vehicles? A preliminary study based on factor analysis.	Vehicle design, including user experience, performance, and aesthetics, is the most influential factor in the purchase of BEVs in China, followed by national awareness (support for local industry and environment).
Williams, B. D. H., & Anderson, J. B.	2023	Williams and Anderson (2023)	From Low Initial Interest to Electric Vehicle Adoption: “EV Converts” in New York State’s Rebate Program	Initially uninterested EV buyers (Converts) prioritized the rebate, downplaying social or technological aspects. Being female was associated with this conversion.
Williams, B. D. H., & Anderson, J. B.	2024	Williams and Anderson (2024)	Expanding Electric Vehicle Adoption in Disadvantaged Communities	EV adopters in disadvantaged communities prioritize adding EVs to the home, non-Tesla EVs, purchases rather than leases, charging, environmental impact, not energy independence. Race/ethnicity, incentives, and knowledge were not differentiating factors.
Wolbertus, R., & van den Hoed, R.	2020	Wolbertus and van den Hoed (2020)	Plug-in (Hybrid) Electric Vehicle Adoption in the Netherlands: Lessons Learned	Sales policies in the Netherlands drove the early adoption of plug-in hybrid electric vehicles by influencing the demand for public charging stations. The focus was on financial incentives and the creation of a robust charging infrastructure.
Zarazua de Rubens, G., Noel, L., Kester, J., & Sovacool, B. K.	2020	Zarazua de Rubens et al. (2020)	The market case for electric mobility: Investigating electric vehicle business models for mass adoption.	EV vendors need technical expertise, a consultative approach, adaptability to new sales models, and the ability to customize solutions for specific customers.
Zhang, X., & Zhao, C.	2023	Zhang and Zhao (2023)	Resale value guaranteed strategy, information sharing, and electric vehicles adoption.	Resale anxiety affects electric vehicle adoption. A resale value guarantee (RVG) can incentivize sales by mitigating this anxiety and increasing supply chain profits.
Zhou, Y., & Li, S.	2018	Zhou and Li (2018)	Technology Adoption and Critical Mass: The Case of the US Electric Vehicle Market.	The interdependence between electric vehicle adoption and charging stations creates critical mass constraints in many markets, hindering their expansion. A targeted subsidy policy would be more effective than the current one.
Zhuge, C., Wei, B., Shao, C., Dong, C., Meng, M., & Zhang, J.	2020	Zhuge et al. (2020)	The potential influence of cost-related factors on the adoption of electric vehicle: An integrated micro-simulation approach.	PHEV subsidies drive PHEV sales, but fuel prices have little macro influence on EV adoption. At the micro level, they affect the spatial distribution of emissions, electricity demand, and the location of charging points. Purchase price is more influential than fuel cost.
